# Co-designing Urban Living Solutions to Improve Older People’s Mobility and Well-Being

**DOI:** 10.1007/s11524-018-0232-z

**Published:** 2018-04-11

**Authors:** Steve Cinderby, Howard Cambridge, Katia Attuyer, Mark Bevan, Karen Croucher, Rose Gilroy, David Swallow

**Affiliations:** 1 University of York, York, UK; 2 Stockholm Environment Institute, Environment Department, University of York, York, UK; 3 Department of Geography, University College London, London, UK; 4 Centre for Housing Policy, University of York, York, UK; 5 Planning and Policy, School of Architecture, Planning and Landscape, Newcastle University, Newcastle, NE1 7RU UK; 6 The Paciello Group, 20-22 Bedford Row, London, WC1R 4JS UK

**Keywords:** Urbanisation, Older people, Mobility, Well-being, Active ageing

## Abstract

Mobility is a key aspect of active ageing enabling participation and autonomy into later life. Remaining active brings multiple physical but also social benefits leading to higher levels of well-being. With globally increasing levels of urbanisation alongside demographic shifts meaning in many parts of the world this urban population will be older people, the challenge is how cities should evolve to enable so-called active ageing. This paper reports on a co-design study with 117 participants investigating the interaction of existing urban spaces and infrastructure on mobility and well-being for older residents (aged 55 + years) in three cities. A mixed method approach was trialled to identify locations beneficial to subjective well-being and participant-led solutions to urban mobility challenges. Spatial analysis was used to identify key underlying factors in locations and infrastructure that promoted or compromised mobility and well-being for participants. Co-designed solutions were assessed for acceptability or co-benefits amongst a wider cross-section of urban residents (*n* = 233) using online and face-to-face surveys in each conurbation. Our analysis identified three critical intersecting and interacting thematic problems for urban mobility amongst older people: The quality of physical infrastructure; issues around the delivery, governance and quality of urban systems and services; and the attitudes and behaviors of individuals that older people encounter. This identified complexity reinforces the need for policy responses that may not necessarily involve design or retrofit measures, but instead might challenge perceptions and behaviors of use and access to urban space. Our co-design results further highlight that solutions need to move beyond the generic and placeless, instead embedding specific locally relevant solutions in inherently geographical spaces, populations and processes to ensure they relate to the intricacies of place.

**Electronic supplementary material:**

The online version of this article (10.1007/s11524-018-0232-z) contains supplementary material, which is available to authorized users.

## Introduction

Globally, our societies are becoming increasingly urbanised with the United Nations (UN) reporting that already the majority of people live in urban settings with predictions this will rise to 66% by 2050 [1]. These projections indicate another 2.5 billion people will be added to urban population by the middle of this century.

Alongside increasing urbanisation is a demographic shift with a significant ageing of the population projected for most regions of the world. In Europe, the UN predicts that by 2050, 34% of the population will be over 60 years old [2]. In the UK, these changes mean that by 2040, nearly one in seven people is projected to be aged over 75 [3].

These combined trends entail that our urban spaces will need to evolve and adapt to the needs of older residents. This challenge is central to the concepts of ‘Age Friendly Cities’ [4] which looks at how urban spaces can be reconfigured (both physically and in terms of service delivery) to enable accessibility and inclusion encouraging active ageing.

The concept of active ageing relates to enabling participation in social, economic and civic life and maintaining well-being through creating opportunities for older people to undertake meaningful and engaging activities to facilitate autonomy and independence [5, 6]. Well-being can be defined in relation to positive functioning associated with social and place relationships, coping strategies and environments (both social and physical) that empower [7]. Well-being encompasses hedonic functions such as pleasure attainment and pain avoidance, and eudemonic linked to a meaningful existence related to personal functioning (within individuals own mental and physical constraints) [8]. Health intersects with well-being in the World Health Organisation definitions of ‘complete physical, mental and social well-being and not merely the absence of disease or infirmity’ [9].

Mobility can be seen as a key aspect of active ageing and enabling participation and autonomy into later life [10, 11]. One facet of mobility is ability to move through physical space [12]. Remaining physically active has been linked to many positive physiological and psychological benefits [13]. Similarly, sustaining physical mobility within communities into old age enables the maintenance of meaningful social interaction [14]. The physical action of moving, particularly walking, but potentially through other forms of motion, has been linked to the concept of ‘therapeutic mobilities’ enabling well-being benefits alongside other health gains [15].

An active ageing mobility focussed approach to promote well-being has been defined as including two key goals:

1. Making cities age-friendly to promote the well-being and social involvement of older residents thereby helping to keep cities thriving, and
2. Not just “elderly friendly” city, instead measures to enable mobility should enhance the independence of a cross-section of society [16].

The need to improve mobility options relates to the findings that older people who live in unsafe environments or areas with multiple physical barriers are less likely to get out and therefore more prone to isolation, depression, reduced fitness and increased mobility problems [17]. The nature of physical environments also influences well-being [18, 19] through salutogenic effects [20] that can mitigate causes of ill health. Recent reviews have advocated the use of relationship-centered approaches to well-being that takes into account the totality of the environment including physical infrastructure but also the actions and behaviors of other users of the space [21]. This includes looking at differential needs for physical space amongst people which vary temporally (across short-term changes in daily roles or long-term, across a life-course). Different landscape settings provide varying degree of cognitive restoration associated with well-being partly dependent upon people’s existing quality of mental health [22].

Buffel et al. [23] have argued that focussing upon ‘what are the actual opportunities and constraints in cities for maintaining quality of life as people age?’ as a better starting point for understanding the complex interrelationship between urban living and ageing [24] than imagining the ideal conurbation. This identification of actual opportunities links to the need to improving participation in urban planning identified in the UN Sustainable Development Goals. A goal target is by 2030 to provide safe, affordable, accessible and sustainable transport systems for all with special attention to the needs of those in vulnerable situations which includes some older persons.

One approach to enable improved engagement grounding developments in the reality of urban residents experience is through co-design [25]. This engagement is intended to lead to the incorporation of a wider range of perspectives [26] and result in the identification of innovative solutions that better reflect users’ self-identified needs [27] within the constraints of the existing urban fabric. Using a mixture of methods and tools [28] has been highlighted as a way of facilitating participants with differing cognitive strengths to make contributions [28, 29].

This paper reports upon a 3-year co-design study identifying options to promote mobility for older residents. Key questions investigated by this study reported upon here are the following:

1. For older people, what are the diversity of factors linked to qualities of urban environments associated with improving or compromising mobility and associated well-being?
2. Can we identify urban assets that provide salutogenic environments for older people?
3. Can we identify co-designed solutions for an age-friendly city which are also beneficial to a wider cross-section of urban residents?

## Methods

**Fig. 1:**
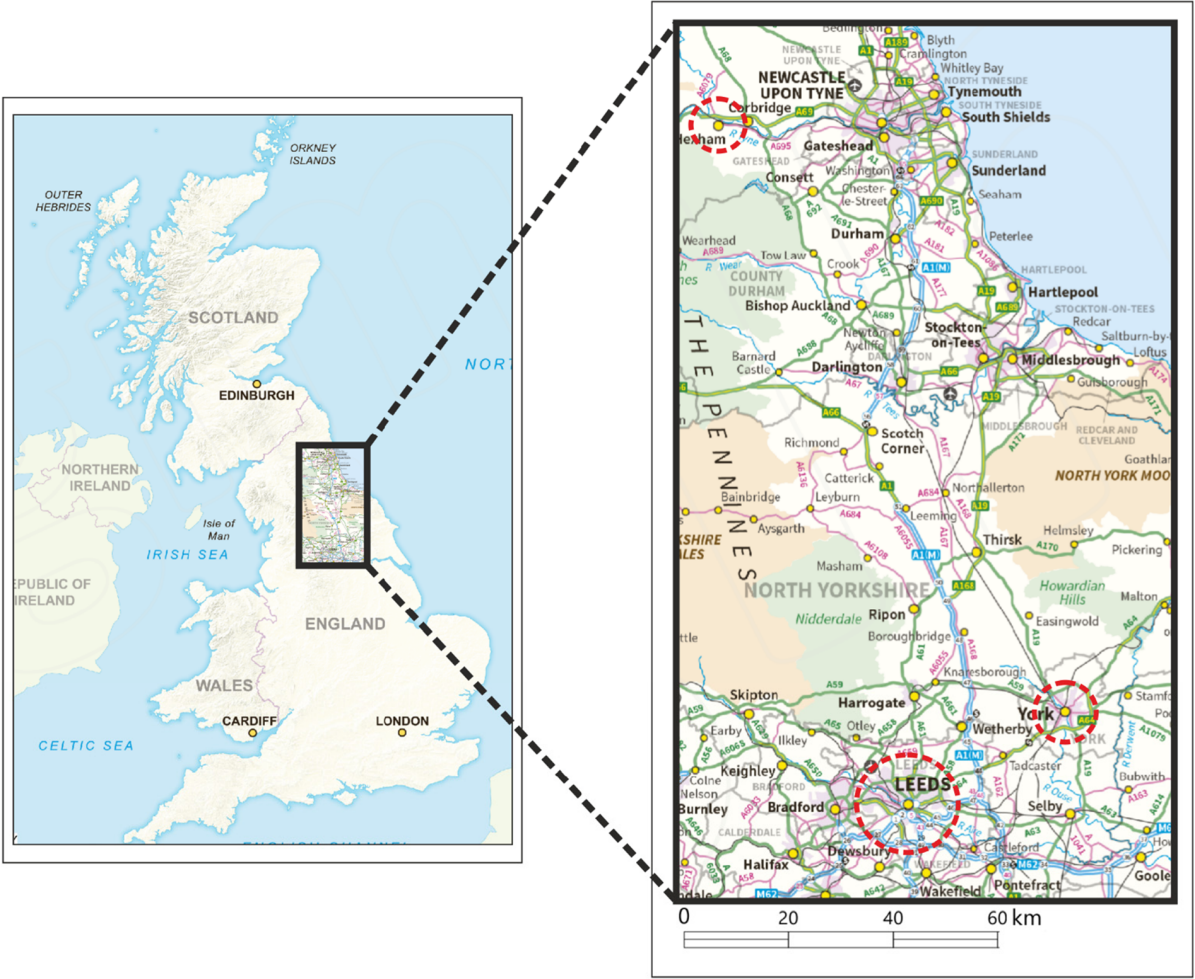
Co-motion case study locations

Co-design activities were undertaken in three case study locations (see Fig. 1) representing a cross section of typical conurbations ranging from Hexham, a small rural town (population 13 K); York, a medium sized city (population 205 K); and Leeds, a large metropolis (population 787 K). UK Office of National Statistics indicate these locations have a transect in terms of their demographic profiles with 12% of Leeds population being non-white; York having an 89% and Hexham 95% white British population. York and Leeds have similar percentages of their populations 65 years and older at 16.8 and 15.6%, whereas Hexham has a higher proportion with 25.4% falling in this age bracket. The sites included a diversity of built environments whose design, topography and infrastructure presented a range of mobility challenges and opportunities. Our participant population sample was chosen to represent a spectrum through the ageing life course from 55 years onwards. This age range was chosen to capture mobility issues related to transitions in social circumstance, health, income and mobility incentives [30]. Participants were recruited using a mixture of methods ranging from leafletting, adverts, talks at older people’s groups and social media to encourage a cross-section of participation from across the case study sites.

Whilst numerous visions of transitions exist, these are often abstract and placeless, sometimes disguising the geographical processes that potentially underpin such transformations [31]. By missing out on the geographical aspect, these fail to take into account how transitions will manifest differentially in place and how the intricacies of place may impact upon such transformations [32]. Our participants were therefore located in specific places to address the spatial specificity of transitions and mobility.

### Co-design Data Collection

Our co-design approach utilised mixed methods [33, 34] incorporating participatory mapping, photo diary elicitation and individual interviews. Different participants were included in the three activities. Ethical approval for the project was obtained via the Social Policy and Social Work Ethics Committee, University of York, and all participants gave their informed consent to take part in the research.

#### Participatory Mapping

During individual interviews participants were asked to list journeys by mode, for different purposes (shopping, leisure, healthcare, socialising), and rate them according to the ease or enjoyability of the trip. Based on these self-reported ratings, a subset of the more problematic journeys was selected for detailed investigation. The selection criteria included how frequently the trips were made, how severe the problem and whether they included issues in the city-center (to enable comparisons between participants in a shared space). Touch screen-enabled PCs were used for collecting journey data spatially and digitally using Edina Digimap mapping toolbox to allow participants to view and interact with scale dependent mapping (from 1:1 M for national overview to 1:1250 for building and land boundaries). Along their routes, participants marked challenging locations (causing the journey to be identified as problematic) in terms of mobility or well-being (alongside positive spaces giving benefits or easy to traversable). For each challenge highlighted, participants were asked to identify solutions that could overcome the issue improving their mobility or well-being. Solutions were not pre-determined but rather open-ended based on participants’ knowledge, experience and imagination.

#### Photo Diary Elicitation

Participants were provided with cameras and asked to photograph anything affecting their travels (positively and negatively) during a 2-week period. During a follow-up interview, they described their images in relation to mobility or well-being effects [35].

#### Individual Interviews

People aged 55 and older were recruited who had recent (within 12 months) experience of one or more critical life transitions [30] such as retiring, starting to live alone or starting to use a mobility aid. During face to face interviews, participants explored the purpose of regular trips, preferred travel mode, practical challenges and importance. They also talked about their transition impacts on ‘getting out and about’. We also queried what would make remaining mobile easier for people in their situation.

### Mobility and Well-being Factor Identification

#### Qualitative Analysis

Transcripts from all three elicitation methods were entered into QSR NVivo for analysis and coding using a grounded theory approach [36]. Specific mobility barriers and identified well-being benefits were collated. Coding was undertaken by one researcher in discussion with a second independent researcher to validate emerging connections. Resulting data was coded in terms of the volume of comments related to specific themes and the number of participants commenting on that factor. These factors and their weightings were extracted and imported into Gephi (open-source graphing software) for visualisation.

#### Spatial Analysis

**Fig. 2:**
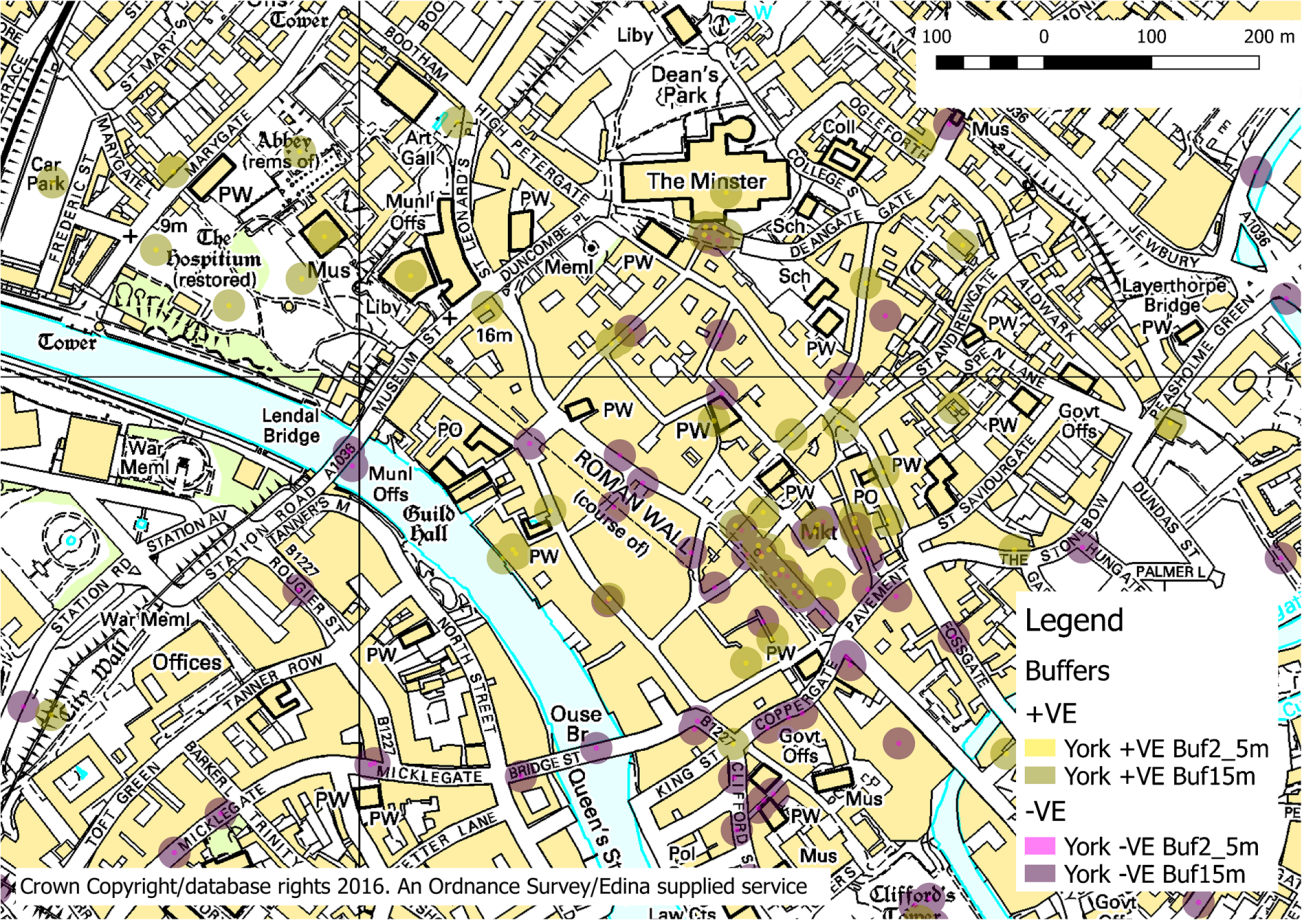
Buffered positive and negatively associated locations identified by participants used in spatial analysis

To explore in depth whether we can determine salutogenic environmental factors, York was used as a case study city where spatial locations of beneficial and problematic spaces identified by older people were combined into a GIS database (in Q GIS). The binary (positive, negative) points were converted to circular polygons with a buffer of 2.5 m to identify conditions immediately experienced at a particular position and 15 m for the wider general characteristics of that location (see Fig. 2). These distances were also picked to account for any spatial inaccuracies in the participatory data, e.g. imprecise location of a pavement vs. building edge.

The buffer polygons were intersected with existing publically available spatial attribute data forming a multidimensional typology [37] linked to environmental conditions and derived spatial layers related to factors identified by our participants (e.g. path widths). These intersected attribute data were exported to MS Access database and Excel spreadsheets for post-processing and ultimately imported into SPSS for statistical analysis. The association with positive or negative interaction on older people’s mobility and well-being was investigated using *t* tests to reveal significant differences between these binary sample data.

### Solution Co-design

#### Qualitative Analysis

Our co-design process involved linking identified problem descriptions to participant-generated solutions and combining complementary options. Where no solutions to address an identified problem had been volunteered, we supplemented options derived from literature and web-searches of possible suitable improvements.

This approach was undertaken for each case study area to identify unique solution sets. In rural Hexham due to low-participation rates, the location-specific solutions identified were supplemented with possible generic options emerging from Leeds and York to evaluate their universality.

### Solution Evaluation

A wider survey was used to assess how the solutions identified by older residents would interact with other users urban travel needs. The survey collected respondents’ basic demographic information, their level of agreement with the co-designed solutions and identified alternative options. In addition, an online version of the surveys asked whether the source of the solutions, originating from older people who were residents of the cities, had influenced participant’s responses.

In York, a dedicated website hosting an online survey was developed and promoted using social media channels, personal communication and advertising at project events. In Leeds, a similar website was created with a location-specific dedicated survey.

In addition, an on-street intercept survey was undertaken in Leeds City Market at a pop-up stand over 2 days during June 2016. Two researchers wearing project branded shirts set up a stall with solution options on roll-up banners. Participants were briefed on the research protocol to ensure informed consent, and their survey responses recorded using tablet PCs. In addition, flyers were distributed explaining promoting the website survey to gather further responses. In Hexham, the on-street intercept approach was also used at the local street market in July 2016.

The surveys were evaluated to identify the levels of agreement for specific co-designed solutions and whether these differed by demographic groups. Text comments were analysed to identify the underlying reasons certain preferences emerged and to capture alternative options.

## Results

### Identification of Factors Affecting Mobility and Well-being

**Fig. 3:**
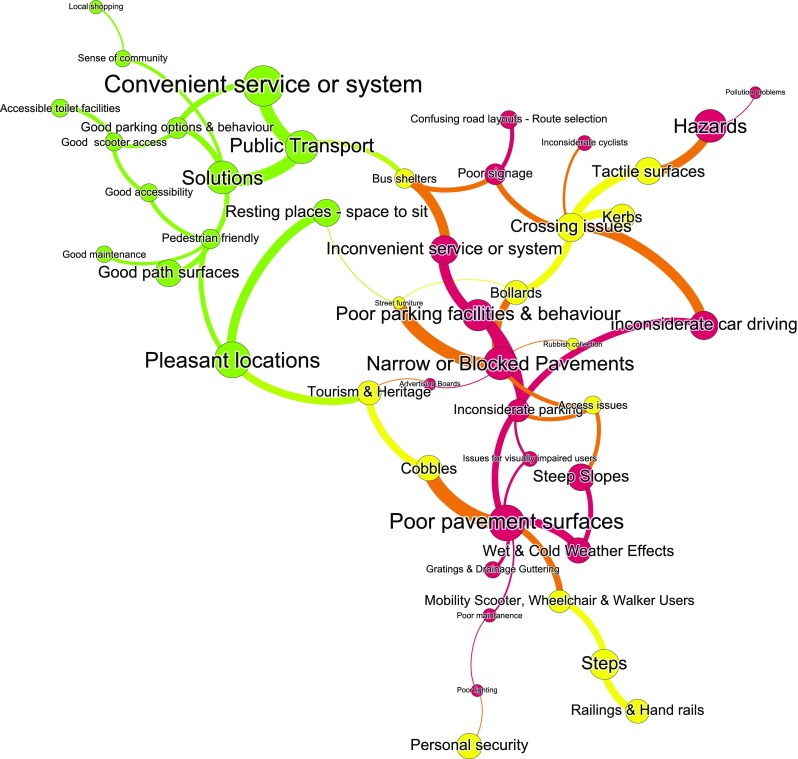
Visualisation of key mobility factors emerging from qualitative data. Nodes size determined by the number of comments related to that factor. Edges determined from qualitative analysis of interview transcripts. Edge width set by the number of participants referring to that factor. Note: Red orange indicates negative factors, yellow indicates mixed factors and green indicates positive factors

Participant numbers can be seen in Table 1. The qualitative data revealed a complexity of factors affecting our ageing population’s mobility and well-being. Visualisation of coded data (see Fig. 3) illustrated the complexity of the interconnectedness and interactions of factors. For example, mobility scooters were problematic for some but represented essential technology for their users; and for these scooter riders the issues were around accessibility of desired destinations.

**Table 1:** Co-design solution identification participant numbers by interaction method

	Age				Location			Gender		
	55–64	65–74	75–84	85 +	Hexham	York	Leeds	M	F	Total
Participatory mapping	7	18	10	4	4	20	15	11	28	39
Photo diaries	11	8	7	0	8	10	8	9	17	26
Interviews	20	19	10	3	2	27	23	18	34	52
Total	38	45	27	7	14	57	46	38	79	117
	32%	38%	23%	6%	12%	49%	39%	32%	68%

Using York for in-depth spatial analysis, it was possible to identify statistical associations between the quality of places and their relationships to encouraging older people’s mobility.

Data from both spatial buffers reveal the well-being benefits of green and blue spaces. Areas frequented by more people (leading to higher rates of personal crime) were also popular destinations for older residents. However, various issues for which spatial data was available reveal aspects of cities that are compromising well-being. Areas with a high density of vehicle traffic leading to particulate pollution [38] were disliked. Spatial analysis supports our qualitative findings that busy city center spaces (where the majority York’s older pre-twentieth century buildings including heritage tourist destinations exist) are problematic. The density of pedestrians also compounds problems associated with narrow pavements. With the larger buffer size, links to air quality and crime disappear (see Table 2). We speculate this could be indicative of the relatively localised and fine-scale differences in these social and environmental variables which the larger buffer size smoothed during analysis.

**Table 2:** *T* test results of statistically significant differences between positive and negative locations

	2.5 m buffer data			15 m buffer data		
Factor	Df (assuming unequal variances)	*T* value	*P* value	Df (assuming unequal variances)	*T* value	*P* value
Area nineteenth Century Buildings	336.769	− 2.687	0.008**			
Area of older buildings (pre twentieth century)				362.341	− 2.977	0.003**
Area of older buildings (pre-twentieth century)	357.281	− 1.750	0.081*	361.802	− 1.821	0.069*
Area of river	335.571	4.752	0.000***	339.929	4.564	0.000***
Area domestic gardens	366.357	3.284	0.001***	366.269	2.715	0.007**
Area green and blue space	216.560	3.295	.001***	277.233	1.726	0.085*
Area-restricted footpath width < 1 m	339.86	− 4.019	0.00***	360.763	− 2.274	0.024**
Crime score	366.883	2.662	0.008**			
Minimum PM_10_	228.679	− 2.131	0.034**			

Note: significance levels *p ⩽ 0.1, **p ⩽ 0.05, ***p ⩽ 0.001

**Fig. 4:**
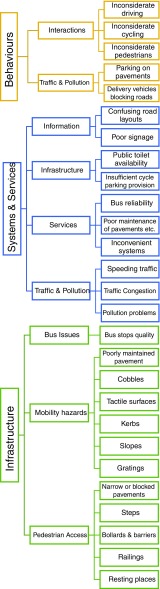
Thematic groupings of factors affecting mobility

Overall similar issues related to the use and quality of environments or infrastructures were revealed by both buffer sizes. From the individual factors supported by the spatial analysis, three critical thematic areas of problems emerged: the quality of physical infrastructure, issues around the delivery, governance and quality of urban systems and services and the attitudes and behaviors of individuals that older people encounter (see Fig. 4).

Physical infrastructure problems included the challenges of poor pavement surfaces interacting with the behavior of vehicle drivers parking on pathways thereby reducing accessibility. Some aspects of pavements were problematic for particular types of mobility. For example, tactile surfaces were mentioned as problematic in general but particularly for those older people using walking aids or wheelchairs [39]. However, for those with visual impairments, these were navigation aids assisting their mobility.

In relation to systems, bus routing, reliability and frequency were experienced problems—particularly as for many older people, buses were a key part of their mobility linked to free travel passes.

A key aspect that emerged was in relation to behaviors which were problems of interactions between different modes of transport in restricted urban spaces. These particularly included shared use paths where cyclists interact with pedestrians. The problem was felt in multiple directions with pedestrians criticising cyclists, older cyclists criticising dog walkers and mobility scooter users having issues with all other modes.

### Co-designed Solutions

**Fig. 5:**
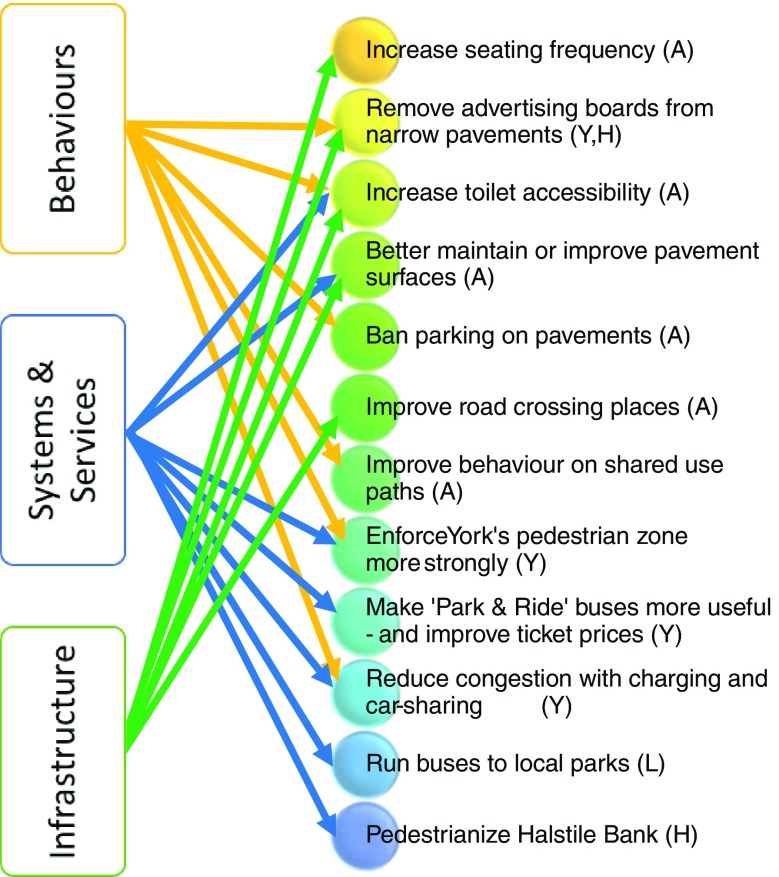
Co-designed solutions linked to thematic groupings (*A* all cities solution, *Y* York-specific solution, *L* Leeds, *H* Hexham)

Obviously, these themes intersect with issues around poor-quality infrastructure being compounded by inconsiderate behavior or sub-optimal delivery of services. For example, better maintenance of surfaces (services and systems) would be complemented by also banning parking on pavements (behaviors) as this was felt to damage paving exacerbating maintenance needs whilst reducing access. In total, 12 co-designed solutions were identified for evaluation (see Fig. 5 below), ten for York and eight for Leeds. The common solutions identified in these two locations were also used in Hexham together with one co-designed by the limited number of local participants.

### Solution Evaluation

The response to solutions varied by option and location; however, some generic patterns emerged. The majority of the co-designed options were well received by a wider sample of the local populations. Many commented that the options would also bring personal benefits or at least not negatively impact upon them, so were supportable if they helped older residents. For example, options to improve seating or ban pavement parking were strongly endorsed.

Options that would affect vehicle use proved more divisive. In York, options introducing road charging inside the inner ring road received the lowest support with only 46% endorsement. Similarly in Hexham, an option to pedestrianise a road into the town center (Halstile Bank) proved controversial and split opinions. Options affecting business behavior were also questioned in terms of implementation and revenues impact. The removal of advertising boards in narrow streets and allowing non-patrons to access toilets in retail outlets fell into these categories.

Participants ranking of solutions also revealed some commonalities but again identified differences based upon varying local conditions (see Table 3). The rankings were useful for differentiation as they forced people to move beyond general endorsement or neutrality toward solution options.

**Table 3:** Survey participant top 3 ranked co-designed solution options. Note ranks are based on weighted scores (weight of 3 for top option, 2 for second choice, 1 for third)

Solution	York	Leeds	Hexham
Enforce York’s pedestrian zone more strongly	1		
Maintain pavement surfaces better	1	1	1
Increase toilet accessibility	3		
Improve road crossing places		2	
Ban parking on pavements across the town		3	2
More seating in city center and shopping centers			3

York had a joint first and hence no second

Assessing if co-designing solutions with older local residents had resulted in their greater relevance or acceptability revealed mixed findings. 32% (*n* = 42) of respondents agreed that having solutions generated by older people had influenced their responses with qualifying comments indicating this influence being universally positive. Similarly, 34% (*n* = 44) indicated that having solutions generated by fellow residents had positively influenced their acceptability. Neither of these results was statistically significant when tested by chi [2].

## Discussion

### Challenges of Co-design

Our study indicates that whilst co-design involving local residents may improve the acceptability of some urban realm changes [32], difficult or challenging options will still meet resistance from the wider population. Solutions that aim to restrict embedded mobility behaviors such as car use, parking or actions in shared spaces generated the most diverse cross-section of responses and resistance.

Additionally during feedback meetings, we were questioned on whether the co-designed solutions were not challenging or radical enough. The high acceptability of the proposed solutions to a wider cross-section of participants supports that this may have been the case. However, it could also be taken as an indication that radical change in cities is not desirable to older people or required for their well-being and mobility.

### Complexity

Our findings indicate the complexity of issues related to mobility and connections to well-being. Our data support findings on physical infrastructure-related barriers or enabling characteristics identified in other studies [40]. However, our results highlight the additional complexities of urban mobility and well-being issues intersecting with the critical juncture of infrastructure, service design and the interaction with people’s behavior, either enabling and overcoming problems for older users or else undermining well-planned services and compounding issues of poor facilities. This reinforces the need for policy responses that may not necessarily involve design or retrofit measures, but instead might challenge perceptions and behaviors that are deemed unacceptable in their impact on the mobility and independence of others (for example, parking on sidewalks or across dropped curbs).

The factors identified as affecting mobility and well-being have a temporal dimension as they relate to the way older residents are using the city at specific times (diurnally and seasonally). The quality of surfaces that are compounded by weather effects particularly in winter is well understood, but also relates to how street users are prioritised (For example, the issue of delivery vehicles blocking pavements intersects with older people’s mobility due to the timing of these commercial operations).

This finding highlights the need for a holistic approach to developing urban areas to enhance mobility and well-being that combines an understanding of the quality of place which includes the systems and uses that mediate people’s uses of these environments. There needs to be a stronger connection and interaction between the opportunities afforded by infrastructure improvements supporting or being enhanced by better service delivery or behavior change campaigns to improve the utilisation of these resources by older people.

There is also a complexity to ageing and issues older people confront related to differing abilities (physical and cognitive). Particular problems were experienced by mobility aid users or those with specific conditions such as sight loss. These groups identified particular factors and locations problematic to their mobility and well-being not experienced by others without these conditions. Research has highlighted that design features intended to promote mobility for people with specific conditions or impairments may lead to inadvertent barriers for others [41]. The extent to which the needs of diverse groups in later life coincide or diverge in relation to design features in the built environment highlights the difficulty of reconciling competing needs and is an issue that requires nuanced policy and practice responses. Indeed, recent discussions have posited an overlap between factors that are conducive to supporting age friendly communities and wider agendas such as the promotion of liveable cities for all ages, especially in relation to the built environment and health [42, 43]. This overlap suggests that the development of age-friendly features may have shared benefits for other groups within the wider population, in addition to older people [42]. The evidence base on the wider benefits of age-friendly design for the key groups within the general population remains limited, however [44]. The key issue here is the need to improve the evidence base on the health benefits of age-friendly design features that explicitly recognises the benefits and trade-offs for key groups across all ages and to take forward not only our understanding of the differential impact that the introduction of specific design features in the built environment may have on diverse groups, but how the sometimes competing needs of different groups may be discussed and prioritised as part of local agenda setting by communities, policymakers and practitioners. Complexity also means that key factors we have identified are not being captured reliably in official datasets used in urban planning. This includes physical infrastructure (such as the location of benches or the quality of pavements) compounded by an absence of reliable information on the functioning of urban systems (such as toilet availability) or the behavior of other urban users (such as the temporal operations of delivery vehicles). If these issues are not readily identifiable from the datasets, they are not likely to be well-considered in decision-making relating to age-friendly spaces.

### Specificity

Our co-design experiment also indicates that generic off-the-shelf solutions may not lead to potential improvements in the development of age-friendly spaces compared with particular changes that local populations prioritise. In York and Leeds, the solutions tested resulted from local consultation and were specific to those places whilst in Hexham many of the tested solutions were generic options generated in the other two study locations and from recommendations in friendly city guidance literature. For example, improving seating availability was a popular solution in York and Leeds where it had been identified as a local infrastructure concern whereas in Hexham where it was one of the generic options, it was relatively unsupported. The responses from our wider survey indicate that co-design solutions specifically tailored to place, and local experience has particular credibility. This demonstrated that differences in local perceptions and experiences mean blanket rolling out of generic solutions will not necessarily be an effective way of encouraging mobility and enhance well-being. Solutions need to move beyond the abstract and placeless to embed in the specificity of inherently geographical spaces and processes to ensure they relate to the intricacies of place.

At a time of increasingly scarce resources and limited state interventions, discussions on the health benefits of the physical design of the built environment occur within a broader socio-political context. That is, commentators writing from a critical social policy perspective have highlighted that discussions on the development of age-friendly communities are taking place in a context of financial austerity, inequalities in later life, limited state support for urban retrofitting using age-friendly design principles [45, 46] and where the needs of some groups have historically been privileged over others in the design of the built environment [47, 48]. WHO’s age-friendly agenda recognises this latter challenge in its principles for the participation of older people in local civic processes. A key aspect of the WHO’s age-friendly agenda is how to move from generic principles to locality specific approaches that reflect the articulated needs and aspirations of local populations. Both research studies and reviews [49, 50] have highlighted diverse approaches across a number of countries to bring the voices of older people into the potential design and delivery of urban and rural places. Nevertheless, a challenge remains to facilitate and enable discussions across all ages as a way of generating debate at local level that could support how specific priorities are reached. This approach needs to embrace not only an examination of evidence-based health benefits of specific design features that are shared by different groups across the life course, but also a recognition that changes to the built environment may be contested for many diverse reasons [51]. For example, even seemingly benign low-cost solutions such as places to rest can provoke intense opposition where they may be perceived as attracting anti-social elements.

## Conclusions

Our results respond to the call from Musselwhite [52] to look at the wider relationship between mobility and ageing in relation to health utilising transdisciplinary [53] and intergenerational approaches to reveal aspects of mobility experiences that are otherwise hidden [52]. Our approach addresses the need identified by the European Innovation Partnership on Active and Healthy Ageing for tools to characterise the triggers promoting active healthy ageing or conversely lead to increasing inactivity [54] alongside calls to ‘qualify’ conventional official GIS-mapping outputs to promote better decision-making [55]. Our mixed method findings contribute to approaches attempting to represent multiple realities of the same space based upon varying participant experiences, histories, knowledge and agendas. The rich nature of the data we revealed indicates that combining open-flexible approaches (e.g. photo-elicitation) and more constrained methods (participatory mapping) can add particular value to exploratory co-investigation research.

The diversity of mobility needs exhibited by older people means there is no one solution suitable for encouraging universal mobility and a generic age-friendly environment. Our choices create mobility winners and losers [56]. Our findings imply the needs for diversity, offering specific types of users’ route options which give them opportunities to access services and facilities, including recreation and social engagement, but which may offer differing levels of difficulty depending on individual abilities.

This ultimately implies that certain locations will provide mobility challenges, for example historic spaces with narrow busy streets or ‘poor’ surfaces (cobbles etc.). This does not mean that improvements to these locations are never possible rather, that destroying the character of place that make it desirable, encouraging mobility in the first instance may not be the ‘solution’ older people would support or implement.

This approach illustrates a broader need for research to examine how needs of diverse groups in later life coincide with groups across all ages. Our approach can be posited as a way of facilitating discussion and debate at local level that not only indicates potential consensus, but also highlights tensions between users. Future applied research by local practitioners might adopt this method to support priority setting.

## Electronic supplementary material

ESM 1 (DOCX 31 kb)
