# Supplementary material for: Co-designing Urban Living Solutions to Improve Older People’s Mobility and Well-Being
Source: J Urban Health. 2018 Apr 11;95(3):409–22. doi: 10.1007/s11524-018-0232-z (PMC5993707; doi:10.1007/s11524-018-0232-z)
Supplement: Supplementary file 1 — (DOCX 31 kb) [file 11524_2018_232_MOESM1_ESM.docx]

# Supplementary Materials

### Project description

Co-Motion was a three year project (2013-2016) that investigated links between mobility and wellbeing amongst older people. Led by the Centre for Housing Policy, University of York, the research Consortium includes the Departments of Computer Science and Health Sciences and the Stockholm Environment Institute also at the University of York; the Institute for Transport Studies, University of Leeds; the School of Architecture, Planning and Landscape, Newcastle University; the Department of Psychology, Northumbria University; and the Bradford Institute for Health Research, Bradford Teaching Hospitals NHS Foundation Trust. This project was one of seven projects supported by the Engineering and Physical Sciences Research Council’s ‘Design for Wellbeing: Ageing and Mobility in the Built Environment’ programme.

One of the multiple aims of the overall Co-motion project was to evaluate the opportunities and challenges of utilising a mixed-methods approach for identifying benefits, problems and solutions for older people’s mobility in these three case study locations. The activity aimed to assess whether mixed methods could usefully be combined and analysed to generate information on locations and sources of conflict and consensus in a cost-effective way. Also whether they could usefully generate options that would overcome some of the conflicts or barriers that were impacting on older people’s mobility and consequent health and wellbeing.

The three methods evaluated were digital participatory mapping – linked to GIS analysis; photo diary elicitation; and individual (longitudinal) interviews.

### Participation Levels

The profiles of co-design solutions generation participants can be seen in table 1.

##### Table 1. Co-design solution identification participant numbers by interaction method.

|  | Age | | | | Location | | | Gender | |  |
| --- | --- | --- | --- | --- | --- | --- | --- | --- | --- | --- |
|  | 55-64 | 65-74 | 75-84 | 85+ | Hexham | York | Leeds | M | F | Total |
| Participatory Mapping | 7 | 18 | 10 | 4 | 4 | 20 | 15 | 11 | 28 | 39 |
| Photo Diaries | 11 | 8 | 7 | 0 | 8 | 10 | 8 | 9 | 17 | 26 |
| Interviews | 20 | 19 | 10 | 3 | 2 | 27 | 23 | 18 | 34 | 52 |
| **Total** | **38** | **45** | **27** | **7** | **14** | **57** | **46** | **38** | **79** | **117** |
|  | *32%* | *38%* | *23%* | *6%* |  |  |  | *32%* | *68%* |  |

##### Table 2. Co-designed solutions with expanded descriptions and justifications that were used in surveys to identify acceptability to a cross-section of residents.

| **Solution Location** | **Title** | **Description** | **Justification** |
| --- | --- | --- | --- |
| Hexham | Pedestrianize Hallstile Bank | The road is too narrow and steep for the heavy vehicles and large traffic volumes - and is often closed in bad weather. Pedestrianization could reduce town centre traffic - or at least direct it down wider roads. | *Pedestrianizing this road could make the town centre more pleasant improving older people’s wellbeing.* |
| Leeds | More seating in city centre & shopping centres | Increase the number of seats - particularly in the city centre and main shopping centres to allow people to take more regular rests. | *Older people often need to take more rests whilst being active. Knowing there will be frequent suitable seating available especially in the city centre will enable them to remain more active.* |
| Leeds | More pedestrian crossings in areas with high density of older people | Road crossings need to be better sited and more frequent to enable people to cross safely in useful places. | *Older people we surveyed identified that crossings - especially in the city centre - are not ideally placed for pedestrian safety and convenience. They felt that more crossings would allow them to navigate around the city centre more safely.* |
| Leeds | Improve pavement surfaces in city centre – grippier surfaces | Make sure Leeds city centre paving surfaces are grippy - even in wet weather. Improve the maintenance of pavements across the city to reduce cracked, uneven or slippy surfaces. Also remove unnecessary bollards and other trip hazards which older people in particular find dangerous. | *This solution would reduce the risk of older people tripping and falling whilst being active in the city.* |
| Leeds | Run buses to parks – allowing older people to access recreation areas | Make sure buses run from areas with high densities of older people to major parks like Roundhay and Temple Newsam to allow daytime recreation in these green spaces. | *Older people we surveyed said they gained wellbeing benefits from being active in green spaces - but they struggled to access them from some city centre neighbourhoods. This solution would allow more older people to remain active using the city parks.* |
| York | Enforce pedestrianized zones more strongly | No delivery vehicles allowed in the pedestrian area after 10am and before 5pm. Encourage early and late deliveries to prevent the need for vans and lorries on shopping streets in the daytime. | *Delivery vehicles in York's pedestrian zones can be off putting for older people as they may not be able to hear the vehicles coming and can sometimes struggle to get out of the way quickly. This is particularly a problem in Coney street.* |
| York | Reduce rush hour congestion | Reduce the number of cars in the city centre by encouraging car sharing and introducing a congestion charge zone with the the inner ring road. Less cars in the city centre would also allow the buses to be more reliable (by not getting stuck in traffic jams) encouraging their use. | *Older people, like many residents, are frustrated about car congestion in the city centre. They are often also regular bus users and get stuck in traffic whilst using public transport.* |
| York | Make Park & Ride Buses more useful | Outside rush hour allow P&R buses to stop more frequently at accessible locations allowing greater public transport use without incurring the need for extra buses. This solution would increase bus frequency along key routes at little or no-cost. The ticket process for P&R buses should encourage group and family use - and could allow you to hop-on-hop-off more easily. | *Older people we surveyed thought this solution could increase bus frequency on main roads without needing extra vehicles. They also felt ticketing needed to be set to encourage families and groups visiting the city to use the P&R option more frequently.* |
| York | Improve behaviour on shared use paths | Pedestrians, cyclists and dog walkers need to be more aware and respectful of each other on shared paths. Cyclists should avoid pavements as this is particularly frightening for older people. For visitors the demarcation between cycle lanes and pedestrians needs to be clearer - but both groups needs to be respectful of one another. | *Older people we surveyed think the shared use paths are useful but think that behaviour by all types of users could be improved to benefit everyone. They especially highlighted that how to use the paths needs to obvious for visitors and tourists.* |
| York & Hexham | More seating in city centre & parks | Increase the number of seats - particularly in the city centre and parks - to allow people to take more regular rests. | *Older people often need to take more rests whilst being active. Knowing there will be frequent suitable seating available especially in the city centre will enable them to remain more active.* |
| York & Hexham | Crossing points | Road crossings need to be better sited and more frequent to enable people to cross safely in useful places. | *Older people we surveyed identified that crossings - especially in the city centre - are not ideally placed for pedestrian safety and convenience. They felt that more crossings would allow them to navigate around the city centre more safely.* |
| York & Hexham | Improve pavement surfaces and remove bollards | Improve the maintenance of pavements across the city to reduce cracked, uneven or slippy surfaces. Also remove unnecessary bollards and other trip hazards that older people find particularly dangerous. | *This solution would reduce the risk of older people tripping and falling whilst being active in the city.* |
| York & Hexham | Remove Advertising Boards | Remove advertising boards from narrow pavements especially around the city centre. | *These boards can block access for older wheelchair users or those with walking aids and are a hazard for those with visual impairment.* |
| York, Leeds & Hexham | Ban parking on pavements | Ban vehicles from parking on pavements. Less pavement parking may also reduce maintenance with less cracking of slabs. | *Older people in our survey highlighted that this behaviour restricts pavement widths and makes paths inaccessible for many older users - particularly those with visual impairment, walking frames, wheelchairs and scooters - meaning they are forced onto the roads.* |
| York, Leeds & Hexham | Toilet availability | Require all retail businesses (shops, cafés and pubs) to make their toilets accessible for everyone during the working day - not just customers. Clearly highlight on the shopfront if there is an accessible toilet. | *Older people often worry about where they can access a toilet. Making more toilets available to them - without having to then buy something - will encourage them to get out more with increased confidence.* |

Participation levels in the solution evaluation surveys varied across the three case study settings and survey methods as seen in table 3 below. This was partly dependent upon the population size of the community being surveyed (Hexham is significantly smaller than the other two cities), but also related to the type of survey undertaken and their duration. In Leeds 9 responses came from the online survey (11% of the total); 6 in Hexham (20%). There was a bias towards greater participation from women in all three locations with overall two thirds of responses from women. In terms of age profile there was a reasonable distribution with a slight bias towards older people (greater than 55 years) who make up 52% of our sample.

##### Table 3: Overall participation in solution surveys by location and age categories

|  | **York** | | | | **Leeds** | | | | **Hexham** | | | | **Overall** | | | |
| --- | --- | --- | --- | --- | --- | --- | --- | --- | --- | --- | --- | --- | --- | --- | --- | --- |
| Age | M | F | **All** | ***%*** | M | F | **All** | ***%*** | M | F | **All** | ***%*** | M | F | **All** | ***%*** |
| 18-24 | 1 | 7 | **8** | ***7%*** | 3 | 2 | **5** | ***6%*** | 1 | 2 | **3** | ***10%*** | 5 | 11 | **16** | ***7%*** |
| 25-34 | 9 | 15 | **24** | ***20%*** | 2 | 4 | **6** | ***7%*** | 0 | 1 | **1** | ***3%*** | 11 | 20 | **31** | ***13%*** |
| 35-44 | 4 | 14 | **18** | ***15%*** | 6 | 8 | **14** | ***17%*** | 0 | 0 | **0** | ***0%*** | 10 | 22 | **32** | ***14%*** |
| 45-54 | 9 | 8 | **17** | ***14%*** | 5 | 6 | **11** | ***14%*** | 0 | 4 | **4** | ***13%*** | 14 | 18 | **32** | ***14%*** |
| 55-64 | 7 | 13 | **20** | ***16%*** | 3 | 8 | **11** | ***14%*** | 2 | 7 | **9** | ***30%*** | 12 | 28 | **40** | ***17%*** |
| 65-74 | 7 | 22 | **29** | ***24%*** | 7 | 16 | **23** | ***28%*** | 3 | 2 | **5** | ***17%*** | 17 | 40 | **57** | ***24%*** |
| 75+ | 2 | 4 | **6** | ***5%*** | 6 | 5 | **11** | ***14%*** | 3 | 5 | **8** | ***27%*** | 11 | 14 | **25** | ***11%*** |
| **Total** | **39** | **83** | **122** |  | **32** | **49** | **81** |  | **9** | **21** | **30** |  | **80** | **153** | **233** |  |
| ***%*** | ***32*** | ***68*** |  |  | ***40*** | ***60*** |  |  | ***30*** | ***70*** |  |  | ***34*** | ***66*** |  |  |

##### Table 4: Co-design option survey responses

| **Solution Option** | **Location** | **Supportive** | **Neutral** | **Not supportive** | **Total** |
| --- | --- | --- | --- | --- | --- |
| Increase seating | York | 96 | 12 | 5 | 113 |
|  |  | 85% | 11% | 4% |  |
|  | Leeds | 80 | 2 | 2 | 84 |
|  |  | 95% | 2% | 2% |  |
|  | Hexham | 24 | 3 | 5 | 32 |
|  |  | 75% | 9% | 16% |  |
| Remove advertising boards from narrow pavements | York | 92 | 14 | 5 | 111 |
|  |  | 83% | 13% | 5% |  |
|  | Hexham | 19 | 2 | 4 | 25 |
|  |  | 76% | 8% | 16% |  |
| Increase toilet accessibility | York | 67 | 21 | 19 | 107 |
|  |  | 63% | 20% | 18% |  |
|  | Leeds | 73 | 8 | 4 | 85 |
|  |  | 86% | 9% | 5% |  |
|  | Hexham | 24 | 5 | 1 | 30 |
|  |  | 80% | 17% | 3% |  |
| Better maintain or improve pavement surfaces | York | 67 | 21 | 19 | 107 |
|  |  | 63% | 20% | 18% |  |
|  | Leeds | 75 | 8 | 2 | 85 |
|  |  | 88% | 9% | 2% |  |
|  | Hexham | 25 | 5 | 1 | 31 |
|  |  | 81% | 16% | 3% |  |
| Ban parking on pavements | York | 88 | 13 | 6 | 107 |
|  |  | 82% | 12% | 6% |  |
|  | Leeds | 67 | 11 | 8 | 86 |
|  |  | 78% | 13% | 9% |  |
|  | Hexham | 28 | 2 | 0 | 30 |
|  |  | 93% | 7% | 0% |  |
| Improve road crossing places | York | 59 | 43 | 4 | 106 |
|  |  | 56% | 41% | 4% |  |
|  | Leeds | 66 | 11 | 8 | 85 |
|  |  | 78% | 13% | 9% |  |
|  | Hexham | 22 | 4 | 4 | 30 |
|  |  | 73% | 13% | 13% |  |
| Improve behaviour on shared use paths | York | 88 | 16 | 3 | 107 |
|  |  | 82% | 15% | 3% |  |
| Run buses to parks | Leeds | 75 | 5 | 4 | 84 |
|  |  | 89% | 6% | 5% |  |
| Pedestrianize Halstile Bank | Hexham | 10 | 11 | 10 | 31 |
|  |  | 32% | 35% | 32% |  |
| Enforce York's pedestrian zone more strongly | York | 96 | 12 | 5 | 113 |
|  |  | 85% | 11% | 4% |  |
| Make 'P&R' buses more useful - and improve ticket prices | York | 75 | 22 | 10 | 107 |
|  |  | 70% | 21% | 9% |  |
| Reduce congestion with charging and car-sharing | York | 49 | 31 | 27 | 107 |
|  |  | 46% | 29% | 25% |  |

##### Table 5: Positive influence of having solutions generated by older people on survey responses

| Participant Age | | 18-24 | 25-34 | 35-44 | 45-54 | 55-64 | 65-74 | 75+ | Total | *%* |
| --- | --- | --- | --- | --- | --- | --- | --- | --- | --- | --- |
| Female | Strongly Agree | 0 | 0 | 0 | 0 | 0 | 1 | 1 | 2 | *3%* |
|  | Agree | 5 | 5 | 2 | 2 | 6 | 3 | 1 | 24 | *30%* |
|  | Made no difference | 1 | 8 | 9 | 7 | 7 | 15 | 3 | 50 | *63%* |
|  | Disagree | 0 | 0 | 0 | 0 | 0 | 1 | 0 | 1 | *1%* |
|  | Strongly Disagree | 0 | 0 | 0 | 0 | 0 | 1 | 1 | 2 | *3%* |
| Male | Strongly Agree | 0 | 0 | 0 | 2 | 0 | 1 | 0 | 3 | *6%* |
|  | Agree | 0 | 5 | 0 | 2 | 4 | 1 | 1 | 13 | *25%* |
|  | Made no difference | 1 | 5 | 0 | 5 | 3 | 5 | 3 | 22 | *42%* |
|  | Disagree | 0 | 5 | 0 | 2 | 0 | 0 | 0 | 7 | *13%* |
|  | Strongly Disagree | 0 | 5 | 0 | 2 | 0 | 1 | 0 | 8 | *15%* |
| All | Strongly Agree | 0 | 0 | 0 | 2 | 0 | 2 | 1 | 5 | *4%* |
|  | Agree | 5 | 10 | 2 | 4 | 10 | 4 | 2 | 37 | *28%* |
|  | Made no difference | 2 | 13 | 9 | 12 | 10 | 20 | 6 | 72 | *55%* |
|  | Disagree | 0 | 5 | 0 | 2 | 0 | 1 | 0 | 8 | *6%* |
|  | Strongly Disagree | 0 | 5 | 0 | 2 | 0 | 2 | 1 | 10 | *8%* |

##### Table 6: Positive influence of having solutions generated from local people on survey responses

| Local People's Solutions | | 18-24 | 25-34 | 35-44 | 45-54 | 55-64 | 65-74 | 75+ | Total | *%* |
| --- | --- | --- | --- | --- | --- | --- | --- | --- | --- | --- |
| Female | Strongly Agree | 0 | 0 | 0 | 1 | 2 | 3 | 1 | 7 | *9%* |
|  | Agree | 3 | 5 | 1 | 1 | 4 | 4 | 2 | 20 | *25%* |
|  | Made no difference | 3 | 8 | 10 | 7 | 7 | 14 | 2 | 51 | *65%* |
|  | Disagree | 0 | 0 | 0 | 0 | 0 | 0 | 0 | 0 | *0%* |
|  | Strongly Disagree | 0 | 0 | 0 | 1 | 2 | 3 | 1 | 7 | *9%* |
| Male | Strongly Agree | 0 | 0 | 0 | 4 | 2 | 1 | 0 | 7 | *13%* |
|  | Agree | 1 | 3 | 0 | 2 | 2 | 1 | 1 | 10 | *19%* |
|  | Made no difference | 0 | 3 | 0 | 4 | 3 | 5 | 3 | 18 | *34%* |
|  | Disagree | 0 | 3 | 0 | 4 | 0 | 0 | 0 | 7 | *13%* |
|  | Strongly Disagree | 0 | 3 | 0 | 4 | 2 | 1 | 0 | 10 | *19%* |
| All | Strongly Agree | 0 | 0 | 0 | 5 | 4 | 4 | 1 | 14 | *11%* |
|  | Agree | 4 | 8 | 1 | 3 | 6 | 5 | 3 | 30 | *23%* |
|  | Made no difference | 3 | 11 | 10 | 11 | 10 | 19 | 5 | 69 | *52%* |
|  | Disagree | 0 | 3 | 0 | 4 | 0 | 0 | 0 | 7 | *5%* |
|  | Strongly Disagree | 0 | 3 | 0 | 5 | 4 | 4 | 1 | 17 | *13%* |
